# A Treatise on Human Physiology for the Use of Students and Practitioners

**Published:** 1888-10

**Authors:** 


TREATISE ON HUMAN PHYSIOLOGY FOR THE USE OF STUDENTS AND PRACTITIONERS OF MEDICINE. By Henry C. Chapman, M. D., Professor of Institutes of Medicine and Medical Jurisprudence in the Jefferson Medical College, of Philadelphia, etc. Octavo, pp. 995. Lea Brothers & Co., Philadelphia. 1887.

To the author and to the publishers of this work we are due an apology for the lateness of the appearance of this notice. Circumstances entirely beyond our control have delayed the examination and precluded the possibility of forming or expressing any opinion until the present time, and even now we fear that upon some points our expressions may be hasty.

It is at all times a pleasure to the earnest student and to the progressive practitioner to place his hands upon new works, or new edition of old works, from which have been eliminated exploded theories, and in which are presented the results of the latest investigations upon any given subject. It is with such views and feelings we entered upon the examination of the work before us, and we are pleased to find that while such elimination is not complete, it presents a text-book full up with the times.

Indeed, in the literature of some portions of the work, particularly that of the discovery of the circulation of the blood, the author with conciseness and precision has treated the subject more fully than we find in most works of the kind.

In the general arrangement of the work, the author does not differ materially from that made by many who have preceded him. He starts out with the chemistry of the proximate principles, thence passes into food and its digestion and absorption.

This leads naturally into the blood and its circulation, to respiration, and the relations of these functions.

On these subjects the work is fully illustrated, and we think more time and space is given to these physiological apparatus and methods of application than the over-taxed student or busy practitioner will have the opportunity of applying properly.

In connection with the structure of the nervous system is taken up the subject of electricity and magnetism and their application to physiology and medicine. To this subject are devoted the pages from 545 to 622, bringing this department of medical physics up to the date of the publication.

This preliminary leads to the consideration of the brain, spinal cord and the cerebro-spinal nerves, together with the ganglionic nervous system, all of which is brought apace with the advanced theories obtaining at the present date. Following these we find the special senses presented with instructive illustrations and explanations.

The treatise winds up with reproduction. While here the author does not cover so much space as has been occupied by some in similar text-books, he presents our present knowledge in this department in a clear and concise manner. Upon the whole, we are pleased with the production, and think that, as a new candidate for public favor, it compares well with those with which the student of medicine and the practitioners of medicine are more familiar.

The author is a professor of the "Institute of Medicine" in one of the leading schools in this country. We have ever been impressed with the idea that "Institutes of Medicine" embraced physiology, to a certain extent, pathology, with the application of the principles of therapeutia, and we had hoped to find more of this in the work, but, like other text-books of the kind, it is greatly restricted to human physiology. The mechanical execution of the work is in the well-known style of Lea Brothers & Co., who, as evidenced by the typing of the book, realize the fact that the eyes of medical students and reading medical men are worth something.

